# Effects of white noise in walking on walking time, state anxiety, and fear of falling among the elderly with mild dementia

**DOI:** 10.1002/brb3.1874

**Published:** 2020-10-09

**Authors:** Sung‐min Son, Sung‐won Kwag

**Affiliations:** ^1^ Department of Occupational Therapy Joenju Kijeon College Jeonju South Korea; ^2^ Department of Emergency Medical Rehabilitation Service Graduate School of Kangwon National University Samcheok South Korea

**Keywords:** fear of falling, mild dementia, state anxiety, walking time, white noise

## Abstract

**Objective:**

This study aimed to analyze the effects of white noise in walking on the walking time, state anxiety, and fear of falling of the elderly with mild dementia.

**Methods:**

Subjects were 32 elderlies with mild dementia, and they divided into experimental group and control group, respectively. In the experimental group, walking program with white noise was applied 3 times a week for 4 weeks. White noise was provided by white noise generator in walking program. In the control group, walking program only was applied. To measure the effect of white noise in walking among the subjects, the walking time, state anxiety, and fear of falling were measured. Walking time was measured by Timed Up and Go test. State anxiety related in walking was measured by Korean version of State‐Trait Anxiety Inventory. Fear of falling was used by Korean Falls Efficacy Scale.

**Results:**

The results of walking time showed the increase in both groups, but the statistically significant difference was not shown. However, the results of state anxiety and fear of falling showed decrease and the statistically significant difference was shown (*p* < .01). In comparative analysis, the statistically significant difference in the results of gate velocity between groups was not shown. However, in the results of state anxiety and fear of falling the statistically significant difference between groups was shown (*p* < .01).

**Conclusions:**

White noise in walking should be induced positively to decrease the state anxiety and fear of falling in walking among elderly with mild dementia. Thus, in their environment, to decrease of state anxiety and fear of falling occurring in walking, the application of white noise in walking situation should be considered to apply for them.

## Graphical abstract

The white noise is an environmental sound which applied in the psychological status by blocking other distracted sound and facilitating to occur the alpha wave. Psychological anxiety related in the performance of the activities is effected positively by the application of the white noise during the activity. As the elderly with mild dementia has a characteristic affecting easily by the environmental noise and stimulation due to the low level of cognition, their gait is easily affected, increasing the risk of falls.

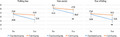

## 1 INTRODUCTION

Dementia is defined as a degenerative clinical syndrome and a progressive disease to be difficult to perform the various activities of daily life significantly due to the cognitive dysfunction (Bowie & Mountain, 1993). Among the elderly with dementia, the elderly with mild dementia accounts for more than 60% and the number has been increased rapidly every year (Beenackers et al., 2011). They have various cognitive dysfunctions. Among their cognitive dysfunction, the dysfunctions of spatial percepttnion, motor praxis, and visual motor organization impact prominently on the increase of the problems in their performance and movement during the activities of daily life (Hernandez et al., 2010).

Actually, their cognitive problems lead not to be aware of the changes and stimulation in the environment and things around them during the various activities. Thus, in their activities, the performance errors and inadequate movements are occurred frequently (Morris et al., 1986). In particular, these impacts of their cognitive dysfunctions during walking act as a factor to increase the risk of falling, collision accidents, and injuries (Barnes et al., 2004) and threaten significantly a safe life and quality of life of them (Hill & Schwarz, 2004). With these impacts, their cognitive dysfunctions also act to lose the direction and way (Morris et al., 1986).

Problems related in their cognitive dysfunction induce the psychological problems such as anxiety, consideration, and fear related in walking and these problems persists for a long time. Particularly, in falling occurred during walking, it leads to a serious disability for the elderly with mild dementia (Sattin, 1992). Accordingly, they are intimidated psychologically and their anxiety and fear are increased remarkably (O'Keeffe et al., 1996). These psychological problems caused by the anxiety during walking effect on the decrease of performance and movement directly. In the walking, they delay or hesitate to start walking and these cause the considerable problems in their daily lives (Barnes et al., 2004).

Among the various stimulations occurred from the environment, white noise is a sound that has a frequency distributed continuously and uniformly over the wide range of the 20–20,000 Hz, and this applied in the form of the natural sound such as wave, rain, and wind, and environmental sound such as car exhaust sound (Sundstom et al., 1994). It plays a role to block the external sound to reduce the detection of the ambient noise and overwhelm other noise in an irregular and open space (Stanchina et al., 2005). As a result, in the performance of activities and task, it can lead to detect effectively the sounds focused by oneself (Soderlund et al., 2007).

In the field of brain science, lots of studies reported that white noise induces the occurrence of alpha waves facilitated in psychologically stable state and it leads the alpha wave to persist for a long time with blocking beta wave facilitated in psychologically distracted state (Afshar et al., 2016). These contribute to improve the performance level by inducing the psychological stability in the performance of activities (Cook et al., 2014). Actually, various studies reported the results from the actions of white noise in the performance of activities (Liu et al., 2007; Söderlund et al., 2010). Also, these actions in learning have been reported (Angwin et al., 2017; Rausch et al., 2014; Vanessa et al., 2014).

In common, these studies argued that the application of white noise in the process of performance of activities and task in learning reduced effectively the psychological anxiety and then the subjects could be able to focus more their tasks and activities (Afshar et al., 2016; Forquer & Johnson, 2007; Stanchina et al., 2005). Following these, this study should be found out the effects of white noise in walking for the elderly with mild dementia who are faced with the problems in walking due to their cognitive dysfunction and have the characteristics affected easily by external environmental stimulation and can increase the risk of falling due to the psychological anxiety and fear occurred in walking.

Nevertheless, there are no studies previously about the effect of white noise in walking on the walking time, state anxiety, and fear of falling of the elderly with mild dementia. Thus, the purpose of this study was to analyze the effect of white noise in walking on the walking time, state anxiety, and fear of falling of the elderly with mild dementia.

## 2 METHODS

### 2.1 Subjects

Subjects were 32 elderlies with mild dementia using a center for dementia in K city, Korea. The selection criteria applied were as follows: They were selected as the subjects diagnosed with mild dementia based of the results of Clinical Dementia Rating (CDR) and Mini‐Mental State Examination—Korean ver. (MMSE‐K) measurement. Also, they were selected as the subjects (a) who have no problems with vision and hearing, (b) who have no neurological or physiological problems of the physical structure to perform the walking, (c) who were not taking antipsychotic medication, and (d) who have desire to participate in this study voluntarily. Potential subjects were recruited from 60 elderlies with dementia, the total number using this center. Among them, 37 elderlies with dementia who are interested in this study hoped to participate in this study. Finally, a total of 30 elderlies were participated in this study and other 7 were excluded based on the selection criteria for subjects.

The process of consent was performed to be based on the ethical standards of Helsinki Declaration. Before consent for them to participate in this study, the enough explanation about the purpose and methods of this study was provided. Then, after understanding about these, they participated in this study voluntarily. Considering their cognitive level and social interaction and communication skills, visual material was used to explain. The consent to the participation was provided in writing by the subjects and a social worker in charge. Additionally, considering the subjects with mild dementia, a social worker in charge cooperated with the process of the consent.

This study was conducted for 4 weeks from 1 May to 30 May 2019.

### 2.2 Study procedure

In this study, the comparative analysis between groups was applied to analyze the effects of white noise in walking on the walking time, state anxiety, and fear of falling among elderly with mild dementia. Before analyzing, the subjects were allocated into experimental group and control group, respectively (*n* = 16) by the stratified sampling method to organize the groups at an equivalent level of the gait velocity, state anxiety, and fear of falling. Additionally, in the process of determining the statistical analysis methods, to analyze the normal distribution based on the central limit theorem, the normality test was performed. As the results of these, the sample distribution was not satisfied with the normal distribution. Thus, the nonparametric test was applied as a statistical analysis method.

Walking program with white noise was applied for the experimental group, and walking program only was applied for the control group. This program was performed 2 times a week for 4 weeks. The program and assessment was conducted, respectively, in the auditorium in the facility. Before the performance of these, distracted environment was arranged and then environment was formed comfortably and stably for subjects. These were performed by an occupational therapist who is a researcher in this study and a social worker in charge was coopered. Assessment was performed individually every month after the walking program.

### 2.3 Walking program with white noise

In this study, walking program was applied between experimental and control groups. Based on the Spring et al.'s study, it was conducted 3 times a week for 4 weeks in an auditorium in the facility. Time of each session was persisted over 100 min and 300 min per week. Prior to start each session, warm‐up and cooldown consisted of the stretching was conducted for 10 min (Sparling et al., 2015).

In the experimental group, walking program with white noise was applied. White noise was provided from the time to start this program to finish in the desk of an auditorium in the facility by Genius Mate P model which is a white noise generator (HDT Co.). In the intensity of white noise, the intensity was set at 40–50 dB which has the highest effects of the application (Pouya, 2016). This intensity was modulated by a noise measurement apparatus. Before providing the white noise, the distracted environment was arranged and the external noise was blocked out completely.

### 2.4 Walking time assessment

To measure the walking time, Timed Up and Go (TUG) test was used. As it is an effective test to measure the comprehensive walking performance level which included dynamic movement and balance, the results of it were used to predict the risk of falling during the walking in daily life (Shumway‐Cook et al., 2000). It measures the total time taking a subject to stand up from an armchair, walk a distance of 3m, turn, walk back to the chair, and sit down by a stopwatch. The total time measurement conducts from the start of walking by the “Go” instruction to the time of sitting again on a chair with the resting on the back of chair correctly and this measures by seconds (Podsiadlo & Richardson, 1991).

In interpretation of results of time, a score of 14 s indicates to have the risk of falls. In a score of 10 s below, it indicates the normal. In a score of 20 s below, it indicates to have good mobility, go out alone, and mobile without gait aid. In a score of 30 s below, it indicates to have problems, not to go outside alone and requires the gait aid. In the results among the ages, the mean value of 60–69 years was 7.9 ± 0.9 s, 70–79 years was 7.7 ± 2.3 s, 80–89 years was 11.0 ± 2.2 s, and 90–101 years was 14.7 ± 7.9 s. The intratester reliability and intertester reliability have been reported as high as 0.98, respectively. The sensitivity and specificity of this test to predict the risk of falling are at 87% (Steffen et al., 2002).

Based on the instruction of TUG test, in this study, the total time of walking 3 m was measured and the results were analyzed. In the process of measurement, subjects also were led to move as quickly as they feel safe and comfortable and there is no time limit. Additionally, subjects were instructed to wear their own regular cloths and shoes. If needed, they were allowed to stop walking and take a rest without it down.

### 2.5 State anxiety assessment

To measure the state anxiety, Korean ver. of State‐Trait Anxiety Inventory (K‐STAI) was used. It is an effective assessment to measure the state anxiety. State anxiety indicates the temporary emotional state caused by tension, anxiety, fear, and distress under the special situation, and it has a characteristic that could be changed in the level (Julian, 2011). This assessment was conducted by the type of self‐questionnaire consisted of 20 subitems. Responses of them assess the frequency of feeling “at this moment” by the following 4‐point scale: (1) almost never, (2) sometimes, (3) often, and (4) almost always. Range of scores for each subtest is from 20 to 80 points, and the higher score indicates the greater anxiety. A cut point of 39–40 has been suggested to detect clinically significant symptoms related in anxiety. The internal consistency alpha coefficients were quite high ranging from 0.86 to 0.95, and Cronbach's alpha of state anxiety was 0.87 (Barnes et al., 2002). Based on the instruction of K‐STAI, in this study, state anxiety was assessed by the numerical value and analyzed.

### 2.6 Fear of falling assessment

To measure the fear of falling, Korean ver. of Fall Efficacy Scale‐International (KFES‐I) was used. It is an effective assessment to measure the fear of falling in daily life. Fear of falling defines as an ongoing concern about falling, which ultimately limits the performance of activities of daily life (Dewan & MacDermid, 2014). It is performed by the type of self‐questionnaire consisted of 16 subitems, which is scored from 1 to 4 points: (1) not at all concerned and (4) always concerned. Range of scores is from 16 to 64 points, and the higher score indicates the greater level of fear of falling. A cut point of 23 has been suggested to have a higher level of fear of falling. The internal consistency alpha coefficients were quite high of 0.90 (Evans & Deaterage, 1969).

### 2.7 Statistical analysis

Collected data were encoded and analyzed by the SPSS ver. 23.0. In determining the statistical analysis method, the Shapiro–Wilk test was used as a normality test. Descriptive statistics was used to analyze the general characteristics of subjects. To analyze the effects of white noise in walking on the gait velocity, state anxiety, and fear of falling among them, Wilcoxon's signed‐rank test was used. In the comparative analysis between groups, the Mann–Whitney U test was used. Confidence level was set at 95% (*p* < .05).

## 3 RESULTS

### 3.1 General characteristics of subjects

Subjects were 32 elderlies with mild dementia. 16 subjects allocated in the experimental and control groups, respectively. Subjects' characteristics are shown in Table 1. In the experimental group, mean age of subjects was 75.42 years, and they consisted of 8 people aged 70–75 and 8 people aged 76–80. Their sex was consisted of 8 males and 8 females. Additionally, their mean height was 168.67 cm in males and 155.33 cm in females and mean weight was 66.83 kg in males and 53.317 kg in females.

**TABLE 1:** Characteristics of the subjects (*n* = 32)

Items	Experimental group		Control group		*Z*	*p*
	Mean	SD	Mean	SD		
Age (*n*, %)						
71–75 years	8 (50)		8 (50)		−0.285	.791
76–80 years	8 (50)		8 (50)			
Average years	75.06	2.82	75.31	3.07		
Sex (*n*, %)						
Male	8 (50)		8 (50)		0.000	1.000
Female	8 (50)		8 (50)			
Height (cm)						
Male	168.50	2.93	168.88	2.53	−0.895	.372
Female	155.63	1.51	157.25	1.49		
Weight (kg)						
Male	67.13	3.23	66.38	2.39	−0.284	.777
Female	53.88	2.69	54.75	3.54		

In the control group, mean age of subjects was 75.25 years and they consisted of 8 people aged 70–75 and 8 people aged 76–80. Their sex was consisted of 8 males and 8 females. Additionally, their mean height was 169.67 cm in males and 156.83 cm in females and their mean weight was 67.17 kg in males and 54.67 kg in females. As the results of the comparative analysis between groups, there was no significant difference in all characteristics between groups within a 95% confidence level (*p* < .05).

### 3.2 Results of walking time

**FIGURE 1:**
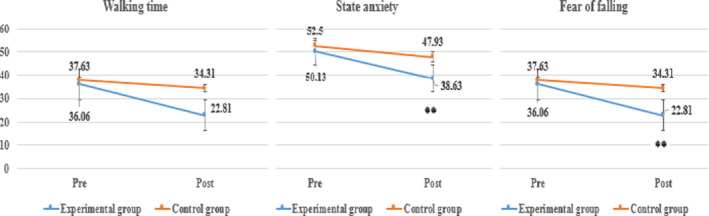
Results of walking time, state anxiety, and fear of falling within group

In the results within experimental group (Table 2, Figure 1), the results of mean walking time showed the decrease at 2.29 s from 15.23 s before the application of white noise in walking to 12.94 s after this. In the results within control group (Table 3, Figure 1), the results of mean walking time showed the decrease at 2.62 s from 16.75 s before the application of white noise in walking to 14.13 s after this. Accordingly, in all groups, the decrease of walking time was shown. However, as the results of the statistical verification, there was no statistically significant difference within each group.

**TABLE 2:** The results in the experimental group

Items	Pre		Post		*Z*	*p*
	Mean	SD	Mean	SD		
Walking time (seconds)	15.23	5.24	12.94	4.01	−1.389	.165
State anxiety (points)	50.13	5.85	38.63	5.74	−2.750	.006**
Fear of falling (points)	36.06	4.02	22.81	5.47	−3.341	.001**

** *p* < .01.

**TABLE 3:** The results in the control group

Items	Pre		Post		*Z*	*p*
	Mean	SD	Mean	SD		
Walking time (seconds)	16.75	6.09	14.13	4.33	−1.624	.104
State anxiety (points)	52.50	4.62	47.93	6.80	−1.823	.068
Fear of falling (points)	37.63	4.73	34.31	7.61	−1.080	.280

Moreover, as the results of comparative analysis between groups (Table 4, Figure 1), in all the pre‐ and post‐test, there was no statistically significant difference even though the variation in experimental group was more than that of control group.

**TABLE 4:** The results between groups

Items	Experimental group		Control group		*Z*	*p*
	Mean	S.D	Mean	S.D		
Walking time						
Pre	15.23	5.24	16.75	6.09	−0.361	.718
Post	12.94	4.01	14.13	4.33	−0.835	.404
State anxiety						
Pre	50.13	5.85	52.50	4.62	−0.488	.625
Post	38.63	5.74	47.93	6.80	−3.214	.001**
Fear of falling						
Pre	36.06	4.02	37.63	4.73	−0.134	.893
Post	22.81	5.47	34.31	7.61	−3.190	.001**

** *p* < .01.

### 3.3 Results of state anxiety

In the results within experimental group (Table 2, Figure 1), the results of mean state anxiety showed the decrease at 11.50 points from 50.13 points before the application of white noise in walking to 38.63 after this. In the results within control group (Table 3, Figure 1), the results of mean state anxiety showed the decrease at 4.57 point from 52.50 points before the application of white noise in walking to 47.93 points after this. Accordingly, in all groups, the decrease of state anxiety was shown. However, as the results of the statistical verification, the results of experimental group only showed the statistically significant difference at the 99% confidence level (*p* < .01).

**FIGURE 2:**
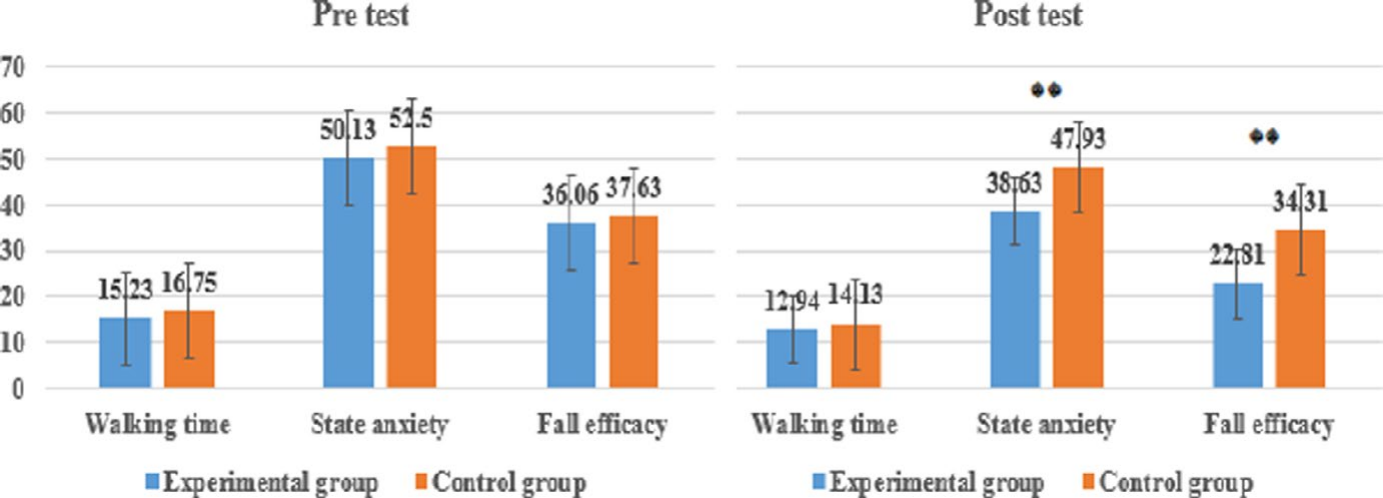
Results of walking time, state anxiety, and fear of falling between groups

Moreover, as the results of comparative analysis between groups (Table 4, Figure 2), only in the post‐test, there was statistically significant difference at 99% confidence level (*p* < .01) and the results of state anxiety in experimental group were 9.30 points lower than that in control group.

### 3.4 Results of fear of falling

In the results within experimental group (Table 2, Figure 1), the results of mean fear of falling showed the decrease at 13.25 points from 36.06 points before the application of white noise in walking to 22.81 points after this. In the results within control group (Table 3, Figure 1), the results of mean fear of falling showed the decrease at 3.22 points from 37.63 points before the application of white noise in walking to 34.41 points after this. Accordingly, in all groups, the decrease of fear of falling was shown. However, as the results of statistical verification, the results of experimental group only showed the statistically significant difference at the 99% confidence level (*p* < .01).

Moreover, as the results of comparative analysis between groups (Table 4, Figure 2), only in the post‐test, there was statistically significant difference at 99% confidence level (*p* < .01) and the results of fear of falling in experimental group was 11.50 points lower than in control group.

## 4 DISCUSSION

This study aimed to determine the effect of white noise in walking on walking time, state anxiety, and fear of falling among elderly with mild dementia. As the results in the walking time, the decrease showed in both group and the results of experimental group were more decrease than that of control group. Participation in walking program acts to increase the participants' walking ability as shown in this study and if white noise is applied in walking, walking ability will be improved more by this (Wuehr et al., 2016). Actually, various studies reported the positive effects of white noise on the performance of activities including the walking.

Generally, the application of white noise plays a role to block the irregular and unpleasant noise generated from the external environment and decrease the detection of this noise (Soderlund et al., 2007). Following these, Ross et al. (2016) reported that white noise acts to improve the walking ability to increase the attention related in movement in walking. These effects also were explained by Wuehr et al. (2016)'s study. They explained the white noise induces to reduce the body sway by focusing to one's body and movement. Accordingly, these act to reduce the performance error and improve walking level (Wuehr et al., 2016).

With these effects, white noise works to prevent the distribution of the attention to decrease performance error and then improve performance level (Ross et al., 2016). Especially, these work more effectively for the people with intellectual disability and the elderly with mild dementia, who are easily affected by external environment stimulation (Stanchina et al., 2005). Therefore, white noise would be good to consider the application of white noise applied as a complementary approach in their environment to improve the level of activity performance for the elderly with mild dementia.

However, in the results of the comparative analysis between groups, the results of walking time did not show the statistically significant difference. Walking is a complex activity included in the integration and coordination among the neurological, physiological, and musculoskeletal system. In particular, the kinetic function and cognitive ability is required vitally to control the posture and movement in order to react appropriately from the external stimulation or environmental changes (Singh et al., 2020). Therefore, it is judged that only the action of white noise could not explain the decrease of walking time in this study.

Accordingly, in order to explain the effect of white noise on walking accurately among elderly with mild dementia, the various studies in the future about the effects of white noise application on the dynamic movements should be performed, with analysis of the neurophysiological and kinetic variables. Additionally, in order to analyze the effects of white noise on walking for them, a long‐term period application of white noise should be applied in the further studies' study design. Therefore, it is judged that this will confirm the applicability of white noise in the performance of activities including walking.

In contrast, the results of state anxiety and fear of falling show the statistically significant decrease in the experimental group and the statistically significant difference in the comparative analysis between groups. These results were demonstrated in various studies reported previously of brain waves. Neurophysiologically, white noise works the facilitation of alpha wave which generates at the state of psychological stability in the brain andmaintain the alpha wave generated for a long time. Through these, the psychological anxiety and discomfort reduce in the performance of activities (Afshar et al., 2016).

Forquer and Johnson (2007) also reported that white noise works to decrease the facilitation of beta wave generated in psychologically distracted state and increase the alpha wave. As the result, the level of performance improved as the subjects were helped effectively to control unpleasant anxiety. Based on these, the results of state anxiety in walking in this study were also decreased. Therefore, to decrease the state anxiety arising from walking, the white noise application should be considered to apply for the elderly with mild dementia as a complementary approach to improve the performance level.

Moreover, white noise has a wavelength of brain waves generated in the state of the psychological stability and sleep (Pouya, 2016). Therefore, the exposure of white noise allows effective control of emotions, and reduces psychological anxiety and especially fear of falling occurring in walking situation, inducing a psychological stability (Barratt & Davis, 2015). These psychological actions along with the effects of white noise, which increase the concentration and attention on the body movements by blocking ambient noise, significantly reduce the fear of falling occurring in walking.

White noise is generated in various forms including the natural or environmental sounds such as rain, wind, car noise, and electronic instrument sound, in daily life, and also, it has a characteristic to be encountered easily and naturally among the various stimulation occurred in the physical environment (Rosalez et al., 2020). Therefore, these characteristics of white noise should be utilized to adequately expose in the process of activities of the elderly with mild dementia. Plus, to decrease the psychological anxiety and fear effectively, in their environment, the application of white noise should be considered.

This study has some limitations. First, as subjects in this study were the elderly with mild dementia, there was a restriction on the number of subjects. Accordingly, it is judged that there are difficulties in generalizing the study results. Therefore, further study suggests increasing the number of subjects to analyze so that the effects of white noise application could be generalized. Second, as this study period was only performed 4 weeks, it was not possible to confirm the effects of white noise on walking time. Therefore, in further study, it is proposed to identify the effects on walking time by increasing the application period of white noise. Additionally, in further study, it is proposed to explain the changes in walking according to the application of white noise by analyzing the neurophysiological and kinetic variables of walking along with the increase of study period are analyzed.

## 5 CONCLUSIONS

The application of white noise in walking effected positively on the decrease of state anxiety and fear of falling among elderly with mild dementia. Based on these, white noise in walking should be induced positively to decrease the state anxiety and fear of falling in walking among elderly with mild dementia. Therefore, in their environment, to decrease of state anxiety and fear of falling occurring in walking, the application of white noise in walking situation should be considered to apply for them.

## CONFLICT OF INTEREST

The authors declared no potential conflicts of interest.

## AUTHOR CONTRIBUTION

All authors were involved in the conception and design of this study, the analysis and interpretation of the data, and drafted and revised the article. SK involved in the intervention of white noise in walking.

### Peer Review

The peer review history for this article is available at https://publons.com/publon/10.1002/brb3.1874.

## Data Availability

The datasets generated and/or analyzed during the current study are from the elderly with mild dementia. Thus, after obtaining the subjects' permission of this study subjects, they are available from the corresponding author on reasonable request.
